# Editorial: Multimodal digital approaches to personalized medicine

**DOI:** 10.3389/fdata.2023.1242482

**Published:** 2023-07-04

**Authors:** Ieuan Clay, Valeria De Luca, Akane Sano

**Affiliations:** ^1^Vivosense Inc., Newport Coast, CA, United States; ^2^Novartis Institutes for Biomedical Research, Basel, Switzerland; ^3^Department of Electrical Computer Engineering, Computer Science, and Bioengineering, Rice University, Houston, TX, United States

**Keywords:** multimodal, machine learning, sensors, editorial, digital measures

## Introduction

Digital health is rapidly evolving and expanding, powering a paradigm shift in evidence generation for clinical development and care delivery (Marra et al., 2020). Driven by advances in cutting-edge biosensors and multi-sensor wearable devices, increasing maturity and acceptance of remote clinical trial models (Izmailova et al., 2021) and digital therapeutics (Stern et al., 2022) and huge growth in computational approaches to processing and interpreting this digital health data, there is great excitement in the field about the possible applications of multimodal digital approaches (Clay et al., 2022). Multimodal data has particular relevance to addressing complex measurement concepts and to development of better personalized treatment, incorporating digital and behavioral phenotyping with molecular endotyping.

New insights enabled by the continuous capture of multimodal data have the potential to drive a deeper understanding of patients' daily life experience (Clay et al., 2023) and more personalized medicines. Novel data science approaches and machine learning applications are pioneering the conversion of multimodal data on physical activity (Lu et al., 2018; Bahej et al., 2019; Mueller et al., 2019; Van Blarigan et al., 2022), sleep (Zhang et al., 2021), vital signs (Jacobsen et al., 2021), cognitive status as well as contextual information, into measures for symptoms and factors associated with health-related quality of life factors such as fatigue (Luo et al., 2020), stress (Sano et al., 2018; Jacobson et al., 2019, 2020), and depression (Jacobson and Nemesure, 2021; Makhmutova et al., 2022). The applications of these measures are diverse ranging from COVID-19 monitoring (Shapiro et al., 2021) to prediction of recovery from orthopedic surgery (Karas et al., 2020).

## Objectives of the Research Topic

Through this Research Topic, we wanted to highlight advancements and challenges specifically related to multimodal digital health data, particularly as applied to developing novel outcomes, patient stratification or predictive models of disease progression. Ultimately, by sharing methodologies, results and data, we aim to lower the remaining barriers to real world adoption of digital health solution.

## Further advances

In this Research Topic, we observed that this early progress is being continued.

Several papers focused on applications of multimodal digital approaches in mental health. Moukaddam et al. presented a mini-review and roadmap, outlining how concepts from computer science and clinical psychiatry can be intersected to highlight a path forward for using digital sensor data to improve how we understand and treat mental illness. Two clinical case studies discussed in the paper proposed and explored specific areas of opportunity. Additionally, two original research papers detailed approaches for detecting and forecasting changes in mental health (Saito et al.; Kathan et al.). Interestingly, both of these papers combined behavioral data from sensors with other inputs (clinical input from medical records and subjective patient input from ecological momentary assessments). The detection of mental health state changes is potentially game changing, particularly in conditions that are often heavily stigmatized as well as being chronic, as knowing when to reach out to someone is hugely powerful.

Continuing with this theme, the Research Topic also contains several other papers focusing on assessing risk through multimodal digital approaches in chronic conditions. For people living with diabetes, physical activity has been shown repeatedly to be beneficial, yet it also carries the risk of inducing hypoglycemic events. Prasanna et al. explored the association of different types and volumes of physical activity, measured through real-world digital measures, with continuous glucose monitoring, laying the foundation for better understanding this phenomenon, reducing risk for diabetes patients and enabling them to better manage their condition. Li et al. presented their work on a clinical data resource focused on enabling the detection and prediction of sudden unexpected death in poorly controlled epilepsy. These rare, but catastrophic, events are the primary cause of mortality in such patients, thus the resource aims to again provide a basis for real-world multimodal digital approaches which could help predict risk and empower patients to better manage their daily lives (Li et al.). The scalability of digital multimodal approaches make them particularly suitable for large scale monitoring, which is important when studying rare events or conditions.

Both Li et al. and Prasanna et al. build on preliminary evidence that alerting systems based on digital measures (i.e., remote monitoring) can help reduce hospitalization in some cohorts (Iqbal et al., 2021). This is an interesting new direction for multimodal digital approaches, with a stronger focus on care delivery than clinical evidence generation (i.e., digital biomarker and COA development; Goldsack et al., 2021).

## Challenges and a path forward

Finally Kristiansen et al. addressed a key challenge in any digital multimodal approach: maintaining data quality as our ability to collect increasing volumes and diversity of data across increasingly uncontrolled settings. Increasing data quality and effectively integrating this data (Clay et al., 2021) will be central to increasing the impact of these approaches, and the growing focus on these issues should be taken as a positive sign that digital multimodal approaches are increasingly being intended for non-exploratory application.

Taken together, we feel that the progress presented in this Research Topic, and elsewhere in the field, is highly encouraging. Continued exploration of what is possible and meaningful to measure through multimodal digital data, further investment into validation and the overcoming of key challenges will expand the boundaries of our ability to assess complex measurement concepts and personalize treatments.

## Author contributions

IC, VD, and AS conceived and wrote the Research Topic concept and this editorial. All authors contributed to the article and approved the submitted version.
